# Triggers and Coping Strategies for Fear of Cancer Recurrence in Cancer Survivors: A Qualitative Study

**DOI:** 10.3390/curroncol29120746

**Published:** 2022-12-03

**Authors:** Xu Zhang, Di Sun, Zhiwen Wang, Nan Qin

**Affiliations:** 1 School of Nursing, Peking University, No. 38, Xueyuan Road, Haidian District, Beijing 100191, China; 2 School of Nursing, Liaoning University of Traditional Chinese Medicine, No. 79, Chongshan East Road, Shenyang 110032, China; 3 Department of Gynecology, Cancer Hospital of China Medical University, No. 44, Xiaoheyan Road, Shenyang 110042, China

**Keywords:** Chinese, cancer survivors, fear of cancer recurrence, qualitative study

## Abstract

Background: Fear of cancer recurrence (FCR) has been demonstrated to be one of the most frequently reported unmet psychological needs among cancer survivors. The aim of this study was to explore and describe the potential triggers and coping strategies for FCR in Chinese cancer survivors. Methods: The study process was conducted using an interpretive phenomenological research method, and Chinese cancer survivors were interviewed face-to-face in a semi-structured interview, using purposive sampling combined with a maximum variance sampling strategy, and the interviews were transcribed, organized, and analyzed by applying Giorgi analysis with the help of NVivo11 software. Results: A total of 10 participants, 4 males and 6 females, were interviewed. Three themes emerged in terms of potential triggers for FCR: (1) intrusive thoughts; (2) disease symptoms; and (3) awaiting medical examination. Two themes regarding positive coping and avoidance coping emerged with regard to coping strategies adopted by cancer survivors when experiencing FCR. Under these 2 themes were 5 sub-themes: (1) seeking medical support; (2) self-health management; (3) spiritual coping; (4) unaccompanied toleration; and (5) attention shifting. Conclusion: FCR as the most common psychological problem for cancer survivors, and it should be given more attention. Early identification and precise intervention for potential triggers of FCR may prevent the emergence and development of FCR. The guidance toward and cultivation of positive coping strategies when cancer survivors experience FCR could be an important direction in future nursing education.

## 1. Introduction

According to Global Cancer Statistics 2020, the global number of new cancer cases is about 19.3 million, and the global number of cancer deaths is close to 10 million; moreover, the number of new cancer cases worldwide is expected to exceed 27 million by 2040, further increasing the global cancer burden [1]. As China is the world’s most populous country, the number of new cancer cases per year in China is almost equal to that obtained the entire American continent (23.7% of the world), and the number of cancer-related deaths is more than the number for Europe (30% of the world), which means that eight Chinese people are diagnosed with cancer every minute. Although the incidence of cancer worldwide is on the rise year by year, with the improvement of medical treatment, scientific and technological progress, and the introduction and application of new anti-cancer drugs, the survival rate of cancer is generally on the rise worldwide, and thus a huge number of cancer survivors has emerged [2]. An individual is considered a cancer survivor from the time of diagnosis throughout the balance of his or her life [3]. Cancer survivors may need to face the possible recurrence or metastasis of cancer at any time during their long-term survival, and because cancer recurrence and metastasis are usually insidious, the fear of cancer recurrence (FCR) is one of the most regularly encountered problems reported by cancer survivors, as well as one of the most common unmet needs among cancer survivors and caregivers [4].

Fear of cancer recurrence is defined as the “fear, worry, or concern about cancer returning or progressing [5]”. In a study by Luo and colleagues investigating the incidence of FCR in 996 cancer survivors from China, it was found that approximately 543 cases (54.52%) reported moderate FCR, and 137 cases (13.76%) reported high FCR, and the results demonstrate that FCR is prevalent among Chinese cancer survivors and should be given attention [6]. Previously, we conducted a meta-analysis study on factors associated with FCR, which confirmed that FCR may be strongly associated with the occurrence of anxiety and depression, increased fatigue, and decreased well-being and quality of life in cancer survivors [7]. In addition, longitudinal studies have shown that while FCR may decline slightly over time, approximately 20% of cancer survivors experience persistently high FCR, and even 10 years after a cancer diagnosis, FCR remains a dominant psychological health problem for survivors [8,9]. Therefore, it is particularly important to explore the triggers and coping strategies of FCR. On the one hand, the identification of potential triggers for FCR in cancer survivors will facilitate the precise implementation of interventions. On the other hand, individual positive coping strategies are beneficial to enrich the content of FCR intervention programs and provide direction for clinical care education.

Most of the current studies on FCR are quantitative [7,8,9]. However, because FCR is recognized as a unique, independent, and multidimensional structure with its own characteristics and mechanisms, targeted interventions need to be constructed. Therefore, the study of FCR from a quantitative research perspective alone may not be adequate. Qualitative research is a way of interpreting people’s worlds, and the feelings, concerns, and experiences expressed by respondents will help offer insight into the overall impact of illness on people, as well as provide a basis for informing psychotherapeutic practice [10]. However, it is worth noting that most of the current qualitative studies on FCR are focused on a single cancer type, and the level of FCR is not assessed before the study subjects are selected [10,11,12]. This leads to one-sided results that do not fully and accurately reflect the reasons for the differences in FCR levels among cancer survivors, which may cause limitations in guiding clinical practice. In addition, qualitative studies on FCR triggers and coping strategies in Chinese cancer survivors have not been reported.

In summary, this study used a qualitative approach to explore the potential triggers and coping strategies for different cancer types and FCR levels through the self-expression of cancer survivors to provide a reference basis for the construction of future intervention programs.

## 2. Materials and Methods

### 2.1. Design

The present study is a qualitative study, which adopts a phenomenological research approach. The core view of the phenomenological philosophical approach is that human experience has a profound meaning, and that it is important to be open and to delve deeply into these experiences in order to understand social phenomena and to make a holistic examination to find the connections between the phenomena and to explore the essence of these life experiences. Phenomenological research methods overlap with the holistic concept in nursing and are therefore widely used in the field of nursing research. The phenomenon of this study is an exploration of the triggers and coping strategies of FCR in cancer survivors in a Chinese cultural context.

### 2.2. Participants

A purposive sampling method was used in this study. Cancer survivors who were hospitalized and treated at the Cancer Hospital of China Medical University were selected. The inclusion criteria were: ① survivors with pathological diagnosis of lung, gastrointestinal, breast, or gynecological malignancies; ② age ≥ 18 years; ③ patients were aware of their diagnosis; ④ clear consciousness and no communication impairment; and ⑤ voluntary participation in this study, with the provision of informed consent. The exclusion criteria were: ① patients with severe cognitive impairment and psychosomatic diseases; and ② patients with low hearing and language expression ability who could not cooperate to complete this study. Based on the inclusion and exclusion criteria, cancer survivors who were able to express their experiences and thoughts in appropriate language were selected.

In the process of purposive sampling, the maximum variance sampling strategy was applied simultaneously, with the aim of enabling the sampled participants to reflect the different situations of FCR experiences more comprehensively and to explore the deeper causes of FCR. This was operationalized by the researcher inviting potential eligible participants to self-complete the Fear of Cancer Recurrence Inventory-Short Form (FCRI-SF) [13], after which participants with large differences in scores were selected for interview based on their scores. The interview sample size was determined based on information saturation (i.e., no new themes emerged), and a total of 10 survivors with different cancer types ultimately completed the interviews.

### 2.3. Data Collection

This study adopted a semi-structured in-depth interview method to collect data, focusing on the trigger and coping strategies of FCR in cancer survivors. The researchers of this study jointly developed a preliminary version of the interview guide before the interview, based on our previous research on factors influencing FCR and discussions with clinical care experts. To ensure the applicability of the interview guide, we selected 2 participants for a pre-interview. After obtaining consent from the participants, one of the study subjects was videotaped throughout the interview. Based on the results of the pre-interview, the researchers repeatedly watched the interview process to summarize the interview technique and revised and improved the interview guide. The researcher explained the purpose and meaning of the interview to the participants before the interview, and also talked to the participants’ families and obtained their consent and trust. Consent was obtained prior to the formal interview, and the interview did not begin until the participant fully understood the study and signed an informed consent form. During the interview, participants were asked to describe whether they experienced the fear of cancer recurrence or progression after being diagnosed, the circumstances under which the fear would arise, the frequency of its occurrence, and the personal coping measures taken when experiencing the fear. The researcher encouraged the participants to speak freely and to actively express their innermost thoughts, while paying attention to obtaining information including language, tone of voice, gestures, and mannerisms, until no new themes emerged, and the interview lasted approximately 20~30 min each. Each interview was recorded and transcribed verbatim.

### 2.4. Analysis

The researchers read the transcribed text carefully several times, and after coding, analyzing, interpreting the phenomena, and refining the elements, initial themes were established. The initially constructed themes were then further condensed to eventually form each higher-order theme and its contained sub-themes. The method of data analysis in this study was Giorgi analysis, which is characterized by first analyzing the essence of the experience of each case, followed by describing the contextual structure of the case and then synthesizing it, resulting in an overall structural description. NVivo 11 (QSR International, Sydney, Australia) software was used as an analytical tool to classify and code the collected data, create nodes, and refine themes.

### 2.5. Rigour

The researcher’s triangulation method was applied to the data analysis phase of this study, where two different researchers applied Nvivo11 software to code the data separately and performed coding reliability tests at the end of coding. The coding reliability in this study was 81%, which is higher than the basic requirement of 70% coding reliability. In addition, a member check was used in this study to ensure the accuracy of the results, and a total of four participants eventually verified the results, and the participants reported that the results adequately reflected the true experience of their FCR. In addition, the researchers were constantly reminded to be alert to biases and preconceptions by maintaining a reflective journal to avoid any influence on the study results.

### 2.6. Ethics

The present study was reviewed by the Ethics Committee of China Medical University (No. 202192) prior to its implementation. The participants selected for the study signed an informed consent form before the start of the study. The principles of privacy and confidentiality were strictly observed during the study. During the interview process, the researcher used only appropriate language to guide, but not judge, the participants’ statements to ensure the impartiality of the study. Interview data were properly protected by the researcher to prevent disclosure. 

## 3. Results

### 3.1. Demographics

A cumulative total of 12 cancer survivors were interviewed in this study, using a purposive sampling method and in accordance with the data saturation principles required for qualitative research. Two of the cancer survivors were pre-interviewed only, for the purpose of interview guide structure debugging and were not included in the formal analysis. Therefore, interview data from 10 cancer survivors were finally included, at which point no new themes emerged to indicate that data saturation had been reached. Full demographics are listed in Table 1. A total of 10 participants, consisting of 4 male and 6 female cancer survivors, including 3 lung cancer, 3 gastrointestinal cancer, 2 breast cancer, and 2 gynecologic tumor patients; the mean age was 51.8 years; the treatment modalities were surgery (4/10), chemotherapy (2/10), surgery and chemotherapy (4/10); the percentage of respondents with fear of recurrence (≥13) was 70% (7/10). Detailed information for each participant is shown in Table S1.

### 3.2. Major Themes

**Figure 1:**
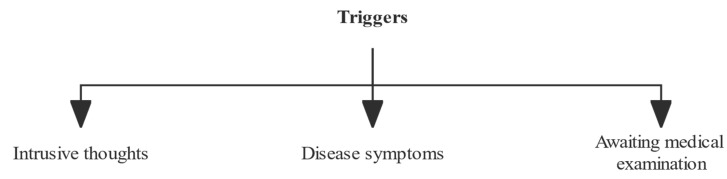
Theme analysis of triggers for fear of cancer recurrence.

**Figure 2:**
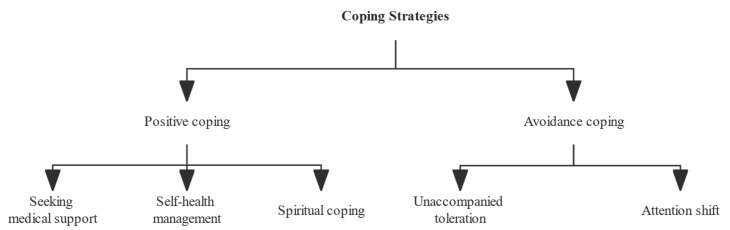
Theme analysis of coping strategies for fear of cancer recurrence.

Separate thematic analyses were conducted on the triggering and coping strategies for FCR in cancer survivors. The study findings regarding FCR triggers reported the following three main themes: (1) intrusive thoughts; (2) disease symptoms; and (3) awaiting medical examination. When experiencing FCR, the coping strategies adopted by cancer survivors can be divided into two themes: positive coping and avoidance coping. Specifically, positive coping is composed of four sub-themes: (1) seeking medical support; (2) self-health management; and (3) spiritual coping. Avoidance coping, on the other hand, is composed of 2 sub-themes: (1) unaccompanied toleration; and (2) attention shifting. The above results are presented in Figure 1 and Figure 2.

#### 3.2.1. Triggers

(1) Intrusive thoughts

Intrusive thoughts are thoughts that enter the individual’s level of consciousness without warning, often with worrisome, disturbing, or bizarre content [7]. Cancer survivors sometimes repeatedly fall into these intrusive thoughts, which can be uncontrollable, causing the individual to experience intense distress.

*“It’s like a dream that comes up in my head every now and then about what happens after the cancer comes back. I want to control it but I can’t control it.”*

*(Participant #4)*

Intrusive thoughts occur at irregular times and are more likely to occur in the dead of night, causing fatigue in cancer survivors by interfering with their rest.

*“I remember a few times, it was one or two o’clock in the morning, and I couldn’t fall asleep. My mind was full of things about tomorrow’s physical examination. The next day, I didn’t have any energy at all, and I was very tired.”*

*(Participant #9)*

The content of intrusive thoughts varies among cancer survivors, but most of them are primarily related to death.

*“I don’t know why, but sometimes I have this image of me dying in my head.”*

*(Participant #1)*

(2) Disease symptoms

The side effects of cancer treatment cause patients to be prone to weight loss, nausea, and vomiting, as well as poor biochemical markers, which may also be considered by cancer survivors to be related to cancer recurrence.

*“When I first got sick, I lost so much weight that I went from 110 to 100 pounds the first time I had chemotherapy. I was really scared.”*

*(Participant #1)*

In addition, cancer survivors often associate abnormal or uncomfortable physical sensations with cancer recurrence.

*“Some time ago I had a little swelling in my neck lymph nodes and I immediately thought could it be a recurrence? Could it have metastasized? I had this kind of association. If there is no major change in the body, then certainly not to consider.”*

*(Participant #6)*

(3) Awaiting medical examination

Cancer requires regular physical examinations after treatment or discharge from the hospital to determine the effectiveness of treatment and the occurrence of recurrence and metastasis. Most of the participants in this interview mentioned their fears while waiting for the results and the doctor’s diagnosis.

*“Last time went to the hospital for review, the doctor told me to do gastroscopy. I was not afraid of the process, but I am very nervous about the result. When I was waiting for the result, I was worried if the stomach cancer had come back.”*

*(Participant #3)*

*“I sat there waiting for the doctor to make a diagnosis based on the CT results, it didn’t take very long, but that time was the most worried and fearful time.*

*(Participant #4)*

#### 3.2.2. Coping Strategies

Positive coping is a way for cancer survivors to use their own resources as much as possible to face their illness, resolve their psychological problems, and return to normal life. Positive coping in this study included the following three sub-themes:

(1) Seeking medical support

Seeking help from a physician out of trust in his or her authoritative knowledge is the most common coping measure for cancer survivors when experiencing FCR.

*“The doctor will tell me how to face my condition properly, so my attitude now is that I will do whatever the doctor tells me to do.”*

*(Participant #2)*

*“I will go to the doctor and listen to their advice. I will do whatever tests the doctor thinks my disease needs to be done, and take the medicine according to the doctor’s order. I trust them more, after all, they are professional.”*

*(Participant #8)*

(2) Self-health management

The uncertainty of cancer recurrence can easily cause cancer survivors to suffer from fearful emotions that can lead to negative outcomes. However, FCR can sometimes motivate cancer survivors to pay more attention to their physical condition, give up bad habits, and develop healthy self-management behaviors.

*“I didn’t pay much attention to my body before, but now I am sick and the doctor told me to keep exercising to strengthen my immune system. Now, as long as my body allows, I will take some time to exercise outdoors almost every day.”*

*(Participant #10)*

*“The doctor told me that both smoking and drinking could aggravate the disease and lead to relapse, so I basically quit smoking and drinking now.”*

*(Participant #5)*

(3) Spiritual Coping

To help oneself cope with FCR by drawing strength from spiritual resources is also a common approach taken by some cancer survivors.

*“I am a Christian, and our church sometimes holds affinity meetings for cancer patients. We prayed and read the Bible together and I felt that it gave me a firm strength to face the disease.”*

*(Participant #7)*

Cancer survivors who avoid coping prefer to use self-closure or distraction methods to force themselves to temporarily ignore or disengage from the existing problem. The details are as follows:

(4) Unaccompanied toleration

Although cancer survivors are suffering from great pain, both physically and emotionally, they usually do not want to show too much, but often tolerate their condition in silence in order to avoid causing worry and to maintain family harmony.

*“I won’t express my distress, because the more I talk about it, the more worried my family will be. It’s better to bear it myself.”*

*(Participant #5)*

(5) Attention shifting

Keeping oneself busy by shifting attention is the most common coping strategy used by cancer survivors when experiencing FCR.

*“I will think about my child and divert my attention, like going out for a walk, getting in touch with the outside world.”*

*(Participant #2)*

## 4. Discussion

To our knowledge, the present study is the first to use a qualitative research approach to explore FCR triggers and coping strategies in Chinese cancer survivors. In contrast to previous qualitative studies on FCR, this study explored in depth the potential triggers of FCR in survivors of different cancer types, increasing the generalizability of the findings [10,11,12]. In addition, we administered quantitative measures of FCR to cancer survivors before the interviews to ensure the maximum differentiation of interviewees, making the information collected on coping strategies more comprehensive and laying an important foundation for guiding clinical practice. It is worth noting that although there were differences in the quantitative results of FCR scores among the participants in this study, all interviewees expressed their worry and fear of cancer recurrence during the actual interviews, which also proved, to some extent, the prevalence of FCR among cancer survivors.

FCR is usually initiated by triggers. In this study, it was determined that the triggering factors may include: intrusive thoughts, disease symptoms, and awaiting medical examination. Seven of the participants mentioned that they had experienced intrusive thoughts. Prolonged and persistent intrusive thoughts can lead to the development of health threats in cancer survivors’ consciousness and increase their doubts about the effectiveness of their treatment or the progression of their disease, which can result in severe uncertainty and psychological maladjustment, if not treated in a timely manner. Our previous evidence-based study found that cancer survivors with high levels of FCR tended to have more frequent and intense intrusive thoughts [7]. The FCR model constructed by Curran et al. also identified intrusive thoughts as a trigger for FCR [14]. Meanwhile, disease symptoms and awaiting medical examination are also important triggers. Due to the stress disorder after cancer diagnosis, cancer survivors are mostly concerned about the changes in their physical condition, and any discomfort or abnormal test results will be regarded as a sign of possible cancer recurrence or progression. In addition, the limited medical knowledge of cancer survivors deepens their suspicion about the disease, increasing the uncertainty of the disease, and eventually develops into FCR. Similar results were reported in a meta synthesis of qualitative studies of FCR [10]. Therefore, the results of this study suggest that timely identification and intervention of FCR triggers may help cancer survivors avoid FCR. It is recommended that clinical staff provide more informational support to cancer survivors to improve their understanding of the disease and reduce their fears.

The choice of coping strategies by cancer survivors may be critical in determining whether FCR ultimately occurs. Participants in this study who adopted positive coping strategies usually chose to seek medical support first, out of trust in the authority of the physician. This may be because the physician can satisfy the patient’s need for information about the disease, reducing uncertainty about the disease or treatment, thus avoiding FCR. The results also emphasize the importance of doctor-patient trust in the medical process, and the establishment of a good communication relationship is considered to be an effective means of increasing doctor-patient trust [15]. Therefore, in view of the increasing importance of the biopsychosocial model of medicine for the communication skills of health care professionals, it is suggested that medical universities and hospitals enhance the training and education regarding communication skills for medical students and clinical medical staff. In addition, self-health management was one of the positive coping styles found in this study. Evidence-based studies have shown that self-health management behaviors can significantly improve the quality of life of cancer survivors, improve pain and fatigue, and also have a helpful effect on psychological stress and sleep conditions [16,17]. Along with the decreasing mortality rate of cancer, cancer has gradually become a chronic disease, and the self-health management of cancer survivors during their survival should receive extensive attention from health care professionals. Spiritual coping is also a coping style adopted by cancer survivors found in this study. Positive spiritual coping has been shown to increase patients’ psychological resilience and reduce negative physical and psychological distress, including as depression, anxiety, and overall stress [18,19]. In addition, spiritual coping has been found to increase social engagement and interaction and improve quality of life, and it may be more effective than religious coping strategies [20]. Therefore identifying the spiritual needs of cancer survivors, improving spiritual coping skills, and taking good spiritual care of these patients may be powerful measures to improve the FCR, possibly becoming a key element or direction for future research.

The majority of participants in this study reported that they did not want to show their psychological or physical pain in front of their families and outsiders, in order to avoid family worries and misunderstandings from outsiders, and thus, they chose unaccompanied toleration. Similar to the results of this study, a tendency to adopt self-tolerance coping was also found in a qualitative study by Levesque et al. of 24 cancer survivors of Chinese descent diagnosed with breast cancer [21]. Tolerating alone is not only detrimental to the recovery of the disease, but in the long run, it may also put a heavy burden on the psychology of cancer survivors, thus increasing the FCR. The reason for this may be, on one hand, related to the traditional Chinese culture that emphasizes family harmony and collectivism. On the other hand, it may be that the general public often lacks proper knowledge about cancer, resulting in low recognition in the external environment and leading to a sense of stigma among cancer survivors. Therefore, it is suggested that the government or relevant departments should increase the popularization of cancer science to correct the misconceptions of the general public, create a favorable external recovery environment for the increasing number of cancer survivors, and construct a health education program with Chinese characteristics to allow cancer survivors to come out from their self-imposed isolation and face their future life bravely. Another common type of avoidance coping is attention shifting. The core purpose of attention shifting is to disengage oneself from the previous conscious activity and engage in a new activity, but the quality of attention shifts during this process is often influenced by the duration, frequency, and nature of the previous activity, which may interfere with the effectiveness of the shift. In contrast, there is evidence that cancer survivors have persistent or worsening chronic mental health problems, such as post-traumatic stress disorder symptoms even 4 years after diagnosis [22]. Therefore, the present study speculated that attention shifting may be more applicable to short-term interventions before and after disease treatment and may have limited effectiveness in addressing the chronic mental problems that develop in cancer survivors. The choice of reasonable coping strategies for cancer survivors cannot be made without the guidance of medical professionals, but at present, health education in China is mainly provided when patients return to the hospital for examination or treatment; therefore, there is a disconnection between hospital and home intervention. In today’s network era, the Internet is widely used in various disciplines because of its spatial independence, easy access to information, and low cost of information exchange. Therefore, providing professional medical information support and guiding patients to adopt the correct coping strategies through an Internet-based continuum of care services for cancer survivors can be a key direction for future research.

There are still some limitations in this study. First, the interviewees in this study were all cancer survivors from the same hospital, and there may be some selection bias. Second, all of the participants in this study were Chinese cancer survivors, and given the potential cultural differences between the Chinese and other world populations, our findings may not be fully applicable to cancer survivors of other ethnicities. Finally, the sample size of this study met the criteria for qualitative research based on the principle of theme saturation, but there is still the possibility that the number of participants included was insufficient, and that individual psychogenesis is a dynamic process, with findings that may vary over time.

## 5. Conclusions

In this study, through the method of qualitative research and in-depth interviews with 10 cancer survivors, the study results revealed potential triggers for FCR in cancer survivors and coping strategies for different individuals. It is recommended that health care professionals identify and intervene early regarding potential triggers of FCR and enhance the education concerning positive coping strategies for cancer survivors to reduce the prevalence of FCR, improve their mental health status, and ultimately achieve a better quality of survival.

## Figures and Tables

**Table 1:** Participant Demographics (n = 10).

Variable		n
Mean age (SD, range)		51.8 (13.8, 30–72)
Gender	Female	6
	Male	4
Employment status	Full-time job	3
	Not employed	1
	Retirement	6
Monthly income, CNY	<2000	3
	2000~5000	5
	>5000	2
Cancer Treatment	Chemotherapy	2
	Surgery	4
	Chemotherapy + Surgery	4
Cancer Type	Lung	3
	Breast	2
	Gastrointestinal	3
	Gynecologic	2
Time since first diagnosis	Less than 3 years	4
	3 to 5 years	4
	Greater than 5 years	2
FCRI-SF score ≥ 13	Yes	7
	no	3

Abbreviations: SD, standard deviation; FCRI-SF, Fear of Cancer Recurrence Inventory-Short Form.

## Data Availability

The data presented in this study are available upon request from the corresponding author.

## Supplementary Materials

The following supporting information can be downloaded at: https://www.mdpi.com/article/10.3390/curroncol29120746/s1, Table S1: Detailed information for each participant.
