# Supplementary material for: Triggers and Coping Strategies for Fear of Cancer Recurrence in Cancer Survivors: A Qualitative Study
Source: Curr Oncol. 2022 Dec 3;29(12):9501–10. doi: 10.3390/curroncol29120746 (PMC9776860; doi:10.3390/curroncol29120746)
Supplement: Supplementary file 1 [file curroncol-29-00746-s001.zip › curroncol-2031810-supplementary.pdf]

Current Oncology

MDPI

Supplementals

Triggers and Coping Strategies for Fear of Cancer Recurrence in Cancer Survivors: A Qualitative Study

Xu Zhang [1], Di Sun [2], Zhiwen Wang [1,*] and Nan Qin [3.]

Table S1. Detailed information for each participant.

Number	Gender	Age	Employment Status	Monthly Income	Cancer Treatment	Cancer Type	Time Since First Diagnosis	FCRI-SF Score
P1	Male	67	Retirement	＞5000	Chemotherapy	Lung	3	5
P2	Female	62	Retirement	＜2000	Chemotherapy+ Surgery	Gynecology	6	19
P3	Female	72	Retirement	2000～5000	Chemotherapy+ Surgery	Breast	10	8
P4	Female	34	Full-time job	2000～5000	Chemotherapy	Gynecology	1	20
P5	Male	42	Full-time job	2000～5000	Surgery	Lung	1	17
P6	Female	50	Retiremen	2000～5000	Surgery	Lung	2	22
P7	Female	53	Retiremen	＜2000	Chemotherapy+ Surgery	Gastrointestinal	4	28
P8	Male	48	Not employed	＜2000	Chemotherapy+ Surgery	Gastrointestinal	3	23
P9	Female	30	Full-time job	＞5000	Surgery	Breast	1	25
P10	Male	60	Retiremen	2000～5000	Surgery	Gastrointestinal	5	10

FCRI-SF score ≥13 indicates the presence of fear of cancer recurrence.
